# Factors Associated With the Perceived Need for Medical Interpreters Among Health Care Professionals

**DOI:** 10.7759/cureus.105226

**Published:** 2026-03-14

**Authors:** Mika Kosugi, Akane Miyamoto, Yue Wang, Francois Niyonsaba, Ai Noda, Kazuya Hara, Naoko Ono

**Affiliations:** 1 Department of Medical Interpreting, Graduate School of Medicine, Juntendo university, Tokyo, JPN; 2 Department of Medical Interpreting, Graduate School of Medicine, Juntendo University, Tokyo, JPN; 3 Faculty of International Liberal Arts, Juntendo University, Tokyo, JPN

**Keywords:** foreign patients, health care professional, international patients, medical interpreter, perceived need for medical interpreter

## Abstract

Background: In recent years, with an increased number of foreign residents in Japan, medical institutions are increasingly required to develop robust systems for accepting and treating foreign patients. However, previous research has not sufficiently investigated factors related to the perceived need for medical interpreters among health care professionals, particularly the relationship between this need and medical professionals’ experience in treating foreign patients.

Objective: The objective of this study was to identify the factors associated with the perceived need for medical interpreters among health care professionals with experience in treating foreign patients, including their willingness to utilize such services and their specific professional roles.

Method: We conducted an online survey between January and February 2024, targeting health care professionals who provided informed consent to participate in the study. A total of 88 individuals with established experience in handling foreign patients were included in the final analysis. To examine these associations, appropriate statistical analyses were conducted. First, the relationships between the perceived need for medical interpreters (categorized as present or absent) and several variables, including basic demographic attributes, awareness of medical interpreting as a profession, prior use of medical interpreter services, and willingness to use medical interpreter services, were examined.

Subsequently, willingness to use medical interpreter services (categorized as present or absent) was treated as the dependent variable, and its associations with professionals’ basic attributes, awareness of medical interpreting as a profession, and prior use of medical interpreter services were analyzed.

The χ² test or Fisher's exact tests were used for categorical variables, and the Mann-Whitney U test was used to compare age and years of professional experience between groups.

Results: For the perceived need for medical interpreters, a statistically significant difference was identified in relation to two factors: age of the health care professional and willingness to use medical interpreter services (p < 0.05). Regarding the willingness to use medical interpreter services, significant differences were observed across a broad range of factors, including the professional’s age, length of professional experience (work history), specific medical occupation (profession type), and prior use of medical interpreter services (p < 0.05).

Conclusion: The findings of this study suggest that the perceived need to use medical interpreters is significantly associated with the health professional’s age and willingness to use medical interpreter services. Furthermore, the willingness to use these services was found to be influenced by age, professional experience, specific profession type, and prior use of medical interpreter services. Importantly, our data indicated that prior experience using a medical interpreter did not directly affect the perceived need for medical interpreters but rather contributed to the intention to use an interpreter in future clinical encounters.

## Introduction

The number of international visitors and foreign residents in Japan has surged recently, with foreign residents reaching a record 3.4 million in 2023 [1-3]. Consequently, Japanese medical institutions face an increasing demand to accommodate foreign patients.

However, a 2017 survey by Japan’s Ministry of Health, Labour and Welfare found that only 50% of hospitals reported accepting foreign patients. Furthermore, among those hospitals, most saw 10 or fewer foreign patients per month [4]. Based on these survey results, despite an expected increase in the number of foreign nationals using medical institutions in Japan, many Japanese hospitals appear to lack sufficient experience with foreign patients.

Medical interpreters support communication between foreign patients and health care professionals. According to the Medical Interpreter Training Curriculum Standards formulated by Japan’s Ministry of Health, Labour and Welfare, medical interpreters are expected to possess the necessary knowledge, vocabulary, abilities, and skills related to health care and public health. In clinical settings, medical interpreters act as linguistic mediators by correctly understanding the speaker’s intent and faithfully conveying it to the listener, thereby enabling effective communication between participants. These professionals are considered essential in health care settings involving foreign nationals, both to protect patients’ rights and to prevent medical errors [5]. Professional medical interpreters are also considered essential for improving patient satisfaction and trust in health care providers. However, a 2024 survey by Japan’s Ministry of Health, Labour and Welfare found that approximately 90% of hospitals do not use medical interpreters [4].

Hamai et al. pointed out that hospitals may be unaware of the risks associated with using untrained interpreters, or they may recognize such risks but rely on ad hoc solutions owing to the low number of foreign patients. However, hospitals that have used dispatched or employed interpreters have reportedly shown a greater willingness to use professional interpreters if the cost were officially covered [6]. Furthermore, according to a survey conducted by the Ministry of Health, Labour and Welfare [4], 85.3% of the 218 medical institutions that used medical interpreters stated that the experience was generally positive. Therefore, experience with using medical interpreters and treating foreign patients may influence health care professionals’ understanding of the need for professional medical interpretation. Although some studies [7,8] have reported the need to educate health care professionals in Japan about the use of medical interpreters and their insufficient recognition of the need for professional medical interpretation, there is limited research investigating factors related to health care providers’ experience with treating foreign patients and their perceived need for medical interpreters. Therefore, this study aimed to identify the factors associated with the perceived need for medical interpreters among health care professionals with experience treating foreign patients, including their willingness to utilize such services and their specific professional roles.

## Materials and methods

Research participants and survey methods

We conducted an online questionnaire-based survey between January and February 2024 through an independent research company (NM), targeting a registered database of approximately 200,000 physicians, 180,000 pharmacists, and 60,000 nurses in Japan. To minimize selection bias, the company randomly invited participants matching the inclusion criteria until the target sample size of 100 responses was reached. Eligible participants were limited to individuals aged 20 years or older who provided informed consent after receiving a thorough explanation of the study objectives and procedures.

The number of responses collected was 100. In this survey, the distribution according to profession was 5,000 physicians, 585 nurses, 500 pharmacists, and 10,376 other co-medical professionals. The total number of respondents was 16,461, and the response rate was 0.60%.

Survey items

Regarding basic demographic attributes, respondents were asked about their sex, age, geographic area of practice, years of experience in health care, native language, highest level of education attained, health care profession, area of specialization, size of their medical institution, and their employment type.

Regarding questions related to knowledge about and experience with medical interpreting as a profession, respondents were asked about their experience in providing care to foreign patients and whether they had ever used a medical interpreter. Depending on the response, additional questions were asked regarding the specific patterns of interpreter use. Furthermore, the survey included questions on the languages used during encounters with foreign patients and medical interpreters, the perceived need for medical interpreters in providing clinical care for foreign patients, and participants’ intention or willingness to use medical interpreting services. The estimated time required to complete the survey was approximately five minutes.

The survey operations were outsourced to NM, and the survey was conducted in accordance with NM’s internal guidelines. Respondents were asked to confirm that they understood the following information before completing the survey: the purpose of the research, the method for withdrawing consent after agreeing to participate, the handling of personal information, and the consultation contact for participants. A checkbox was included to indicate agreement to participate in the research. Respondents who checked this box were considered to have provided informed consent.

To reduce the burden on survey participants, the following compensation was offered: 1,000 JPY was offered to physicians and 800 JPY to other healthcare professionals. Compensation was provided in the form of points equivalent to the stated amounts (1 point = 1 JPY), which could be exchanged by participants for items of their choice, such as Amazon gift cards, through the member platform.

Operational definitions

In this study, the perceived need for medical interpreting is operationally defined as the belief that medical interpreting is necessary in health care settings involving foreign patients. In addition, willingness to use medical interpreter services is operationally defined as the intention to use medical interpreter services when providing care to foreign patients. Health care professionals were classified into three groups: physicians, nurses, and other health care professionals.

Statistical analysis

To clarify factors related to the perceived need for medical interpreters among health care professionals with experience in caring for foreign patients, the analysis was limited to respondents who reported having such experience. This criterion was established to ensure that the findings reflect informed perceptions rooted in actual clinical settings. One respondent whose sex was unspecified was excluded from the analysis.

Appropriate statistical analyses were conducted to examine associations between the perceived need for medical interpreters and multiple variables, including basic demographic attributes, awareness about medical interpreting as a profession, prior use of medical interpreter services, and willingness to use medical interpreter services. In addition, intention to use medical interpreters was analyzed as a dependent variable in relation to the same set of variables.

Categorical variables were analyzed using the χ² test. In cases where any cell had an expected count of less than 5, Fisher’s exact test was applied to ensure statistical rigor. For continuous variables, age and years of professional experience were compared between groups using the Mann-Whitney U test.

To identify which professional groups contributed significantly to overall differences, adjusted residual analysis was performed when significant associations between professions were detected using the χ² test. Adjusted residuals represent standardized values indicating the divergence between observed and expected frequencies; values exceeding ±1.96 were considered statistically significant. A two-sided p-value of < 0.05 was considered statistically significant.

All statistical analyses were conducted using IBM SPSS Statistics for Windows, Version 29 (Released 2022; IBM Corp., Armonk, New York).

Ethical considerations

This study was approved by the Research Ethics Committee of the Faculty of Medicine at Juntendo University (Approval no.: E23-0327-M02; Date of approval: November 25, 2024).

It was explained in writing that participation in this survey was entirely voluntary and that the collected questionnaires would be used solely for research purposes and would not be provided to any external organizations. It was also stated that although the research findings may be presented at academic conferences or published in scientific journals, no personally identifiable information would be disclosed. Furthermore, it was made clear that the principal investigator would take full responsibility for appropriately managing the collected questionnaires to prevent data leaks, contamination, theft, or loss. A checkbox was provided to confirm consent to participate in the study. If the checkbox was selected, this was considered to indicate that respondents had provided informed consent to participate in the survey.

## Results

Excluding one respondent whose sex was unspecified, the remaining 88 participants included 29 women and 59 men. The most common age group comprised individuals in their 40s (n = 31), followed by those in their 30s (n = 30), with a mean age of 45.2 years. Regarding years of professional experience in health care, the largest group had 10 to < 20 years of experience (n = 41, 46.6%), followed by those with 20 to < 30 years (n = 24, 27.3%), with an average of 20.0 years. In terms of professional role, nurses comprised the largest group (n = 29, 33.0%), followed by physicians (n = 26, 29.5%) and other health care professionals (n = 33, 37.5%). As for the type of medical institution, 63 respondents (71.6%) were employed at a hospital (general hospital, university hospital, or national hospital), and 25 (28.4%) worked at a clinic, medical office, or private practice.

Regarding awareness about medical interpreting as a profession, 62 respondents (70.5%) reported being aware of this profession and 26 (29.5%) indicated that they were unaware of this profession. In terms of prior use of medical interpreter services, 59 respondents (67.0%) reported having no experience, whereas 29 (33.0%) had used such services. When asked about the perceived need for medical interpreting in treating foreign patients, 78 respondents (88.6%) answered affirmatively and 10 (11.4%) did not. Regarding the willingness to use medical interpreter services, 74 respondents (84.1%) indicated that they were willing to use such services and 14 (15.9%) answered that they were not.

Table 1 presents cross-tabulation results for the perceived need for medical interpreting. Statistically significant differences were observed for age and willingness to use medical interpreter services (p < 0.05). A marginally significant trend was found for the type of medical institution (p = 0.056). No significant differences were observed for sex, years of professional experience, professional role, awareness about medical interpreting as a profession, or prior use of medical interpreter services (p > 0.1).

**Table 1:** Cross-tabulation results for perceived need for medical interpreting. *Mann–Whitney U test.

Variable	Category	Total (N = 88)	Medical interpreter needed for foreign patients		p value
			Yes	No	
Age (years), median (IQR)		43.5 (38.0–51.5)	42.5 (37.0–50.0)	51.5 (42.0–57.0)	0.041*
Work experience (years), median (IQR)		18.0 (13.0–27.7)	17.5 (13.0–28.0)	20.0 (18.0–26.0)	0.228*
Sex, n (%)	Female	29 (33.0%)	28 (35.9%)	1 (10.0%)	0.156
	Male	59 (67.0%)	50 (64.1%)	9 (90.0%)	
Medical profession, n (%)	Physician	26 (29.5%)	23 (29.5%)	3 (30.0%)	0.189
	Nurse	29 (33.0%)	28 (35.9%)	1 (10.0%)	
	Other	33 (37.5%)	27 (34.6%)	6 (60.0%)	
Medical facility, n (%)	Clinic, other	25 (28.4%)	25 (32.1%)	0 (0.0%)	0.056
	Hospital	63 (71.6%)	53 (67.9%)	10 (100.0%)	
Knowledge about medical interpretation as an occupation, n (%)	Yes	62 (70.5%)	57 (73.1%)	5 (50.0%)	0.153
	No	26 (29.5%)	21 (26.9%)	5 (50.0%)	
Experience using medical interpreters, n (%)	Yes	29 (33.0%)	28 (35.9%)	1 (10.0%)	0.156
	No	59 (67.0%)	50 (56.8%)	9 (90.0%)	
Intention to use medical interpreters, n (%)	Yes	74 (84.1%)	72 (92.3%)	2 (20.0%)	< 0.001
	No	14 (15.9%)	6 (7.7%)	8 (80.0%)	

Table 2 shows the cross-tabulation results for willingness to use medical interpreter services. Significant differences were found for age, years of professional experience, professional role, and prior use of medical interpreter services (p < 0.05). A marginally significant trend was observed for the type of medical institution (p = 0.060). No significant differences were found for sex or awareness about medical interpreting as a profession (p > 0.1).

**Table 2:** Cross-tabulation results regarding intention to use medical interpreters. *Mann–Whitney U test. **Fisher's exact test.

Variable	Category	Total (N = 88)	Intention to use medical interpreters		
			Yes	No	p value
Age (years), median (IQR)		43.5 (38.0–51.5)	42.0 (37.0–49.0)	54.5 (42.0–57.0)	0.014*
Work experience (years), median (IQR)		18.0 (13.0–27.7)	17.0 (12.0–27.0)	20.5 (18.0–32.0)	0.022*
Sex, n (%)	Female	29 (33.0%)	27 (30.7%)	2 (2.3%)	0.130**
	Male	59 (67.0%)	47 (53.4%)	12 (13.6%)	
Medical profession, n (%)	Physician	26 (29.5%)	22 (25.0%)	4 (4.5%)	0.005
	Nurse	29 (33.0%)	29 (33.0%)	0 (0.0%)	
	Other	33 (37.5%)	23 (26.1%)	10 (11.4%)	
Medical facility, n (%)	Clinic, other	25 (28.4%)	24 (27.3%)	1 (1.1%)	0.060**
	Hospital	63 (71.6%)	50 (56.8%)	13 (14.8%)	
Knowledge about medical interpretation as an occupation, n (%)	Yes	62 (70.5%)	55 (62.5%)	7 (8.0%)	0.107**
	No	26 (29.5%)	19 (21.6%)	7 (8.0%)	
Experience using medical interpreters, n (%)	Yes	29 (33.0%)	28 (31.8%)	1 (1.1%)	0.030**
	No	59 (67.0%)	46 (52.3%)	13 (14.8%)	

For the variable of professional role, respondents were categorized into three groups: physicians, nurses, and other health care professionals. A χ² test was conducted to examine the association between professional role and the willingness to use medical interpreter services, revealing a statistically significant difference (p = 0.005). Standardized residual analysis was performed to further examine the relationships among professional roles. Among physicians, standardized residuals were ±0.1 for both groups (willing and unwilling), indicating no significant difference. Among nurses, the residual was 2.9 for those willing to use medical interpreter services and −2.9 for those unwilling to use these services. Among other health care professionals, the residual was −2.9 for those willing and 2.9 for those unwilling to use a medical interpreter, indicating an inverse trend compared with nurses.

## Discussion

As stated in the objective, this study identified several factors associated with the perceived need for medical interpreters, specifically focusing on professional roles and willingness to utilize services.

The results indicated that younger professionals and those with a greater intention to use medical interpreters were more likely to perceive a greater need for medical interpreters. Additionally, a strong association was observed between perceived need and intention to use, suggesting that those who recognize the need for medical interpretation are also more willing to use such services.

In the present study, although approximately 30% of participants had experience using professional medical interpreters, this limited uptake suggests that use remains low, even among those who perceive interpreters as necessary and express a willingness to use them. A nationwide study of 599 Swiss primary care physicians by Fabienne et al. [9] identified the following key barriers: cumbersome organization (58.7%), absent financial coverage (53.7%), and lack of knowledge regarding how to arrange interpreter interventions (44%). Given these findings, it is reasonable to consider that similar organizational and financial challenges may also contribute to low interpreter use in Japanese clinical settings, despite a recognized need.

Based on the survey results, health care providers’ perceived need for medical interpreting was significantly associated with age, but no statistically significant association was found with years of professional experience. This suggests that perceptions of necessity may be shaped more by generational values linked to age than by professional education or clinical experience. A public opinion survey conducted by the Cabinet Office of Japan [10] on environmental development for accepting foreign residents showed that the proportion of respondents who felt such support was necessary (including "somewhat necessary") was highest among those aged 18-29 years, followed by those aged 30-39 years. These results suggest that younger age groups are more likely to believe that actions should be taken to accommodate foreign residents in Japanese society. Accordingly, younger health care providers may also be more likely to recognize the need for medical interpreting within clinical settings, influenced by generational attitudes.

The willingness to use medical interpreter services differed significantly by age, with the younger group reporting a greater willingness to use these services. According to the Japanese Ministry of Internal Affairs and Communications' 2020 Information and Communications White Paper [11], efforts have been promoted in Japan since 2014 to facilitate global and free exchange through multilingual speech translation technology, culminating in the construction of the Multilingual Speech Translation Platform in April 2019. This platform has focused on areas such as tourism, transportation, medical care, and disaster prevention, conducting technical demonstrations aimed at the social implementation of translation services, which has led to the proliferation of various translation services by private companies.

It is possible that young people, who routinely use such technology, have a lower psychological hurdle to overcome in using language assistance. Younger populations may be comfortable using external tools to communicate with people speaking different languages, or they may be more aware of the convenience of such tools than their older counterparts. This suggests that younger people may perceive interpreting not as a specialized intervention but as a practical and easily accessible tool. Therefore, it is hypothesized that younger health professionals have less resistance to the introduction of medical interpreter services and a greater willingness to use these services.

Regarding the willingness to use medical interpreters, a significant difference was observed based on clinical experience (length of service), with the group reporting a greater willingness to use these services having shorter clinical experience. In their systematic review on uncertainty tolerance in health care [12], Yap et al. stated that a provider's lack of experience hinders their ability to address clinical difficulties; conversely, background and experience assist individuals in making well-informed decisions. Considering this background, it is possible that less experienced health care providers are unable to respond well when faced with patients whose language differs from their own and that these providers would be more likely to consider using a medical interpreter as a way to compensate for uncertainty via an external resource, thereby leading to a greater willingness to use interpreter services.

Tohnai et al. [13] reported that whereas expert nurses possess greater reasoning capacity, novice nurses have limited reasoning capacity and thus have fewer options regarding nursing actions. This suggests that the range of actions available in various clinical situations may differ between experienced and less experienced health care providers. Therefore, when encountering patients who have language communication difficulties, experienced health care providers may not rely solely on verbal communication. Instead, they may use various clinical skills, such as non-verbal communication through gestures or drawing diagrams, to communicate with the patient. It is therefore hypothesized that experienced providers have a lower willingness to use medical interpreter services than providers with less clinical experience.

Although we observed certain tendencies across affiliated medical institutions regarding the perceived need for medical interpreting and the willingness to use medical interpreter services, no statistically significant differences were detected in this study. However, the responses were highly skewed, evidenced by the fact that all respondents affiliated with clinics reported a need for medical interpreting. This suggests that the limited sample size likely resulted in insufficient statistical power. Moving forward, comprehensive statistical verification should be pursued in a larger-scale survey.

Regarding the perceived need for medical interpreting, no significant associations were observed among professional categories. However, a difference was noted in the willingness to use medical interpreter services, with all nurse respondents reporting such willingness. Conversely, the number of other health care professionals who stated that they were unwilling to use such services was higher than the expected value based on residual analysis. Ishii et al. [14] pointed out that the greatest stressor faced by nurses when dealing with foreign patients is language difficulties. Nurses’ perceived inability to speak English, coupled with a high frequency of interaction, contributes to this stress. Furthermore, Westbrook et al. [15] reported that nurses spend 37% of their working hours with patients, averaging 3.1 hours of patient contact during an 8.5-hour shift. Because nurses’ tasks are diverse and they have longer patient contact times than other medical professionals, nurses are likely to experience communication difficulties more frequently, potentially resulting in situations where explanations are not effectively conveyed, or the nurse cannot understand what the patient is saying. Izumi [16] indicated that the confusion, hardship, and anxiety experienced by ward nurses when caring for foreign patients are largely influenced by language barriers. Based on this background, it is plausible that nurses expressed a greater willingness to use available medical interpreter services than other survey respondents. Yuu et al. [17] suggested that the heavy reliance of nurses and other medical staff on conversational data obtained from patients is one factor contributing to their demand for a high level of communication skills from medical interpreters. Nurses expect medical interpreters to possess specialized skills to accurately convey a patient’s subtle complaints and detailed medical conditions. This seeking of skills that cannot be replicated by machine translation likely explains why nurses exhibit a greater willingness to use human medical interpretation services compared with other professionals, as these services contribute to the high value placed on the perceived need for medical interpreting.

Ishii et al. [14] revealed that nurses stated that on-site interpretation was not necessary for procedures such as assisting with toileting and measuring vital signs. This may be because the verbal communication used in these situations is relatively standardized, or because the intention can be conveyed through gestures even if words are not fully understood. This suggests that for professionals included in the "other health care professionals" category in this study, such as physical therapists and radiologic technologists, the routine verbal communication used in their work is relatively fixed. Therefore, preparation such as creating precompiled phrasebooks or providing visual communication tools may enable a certain degree of effectiveness in managing foreign patient interactions, potentially leading to a lower perceived need for medical interpreters among such health providers.

Regarding the experience of using medical interpreters, although no significant difference was observed in the perceived need for medical interpreting, a significant difference was found in the willingness to use medical interpreter services. Hattori [18] suggested that many health care professionals in Japanese medical settings are unfamiliar with medical interpreters and consequently cannot understand their roles and practices. This highlights that the profession of medical interpretation is not yet commonly known among Japanese health care providers. Medical interpreters play various roles beyond simple language transfer, including patient advocacy and promoting cross-cultural understanding. However, in a limited interpretation setting, the interpreter lacks the time to explain these roles to the provider. It is therefore difficult for providers to grasp the full extent of a medical interpreter’s importance in foreign patient care unless they personally receive assistance with cultural understanding or observe patient advocacy in practice. Consequently, merely using a medical interpreter may not be sufficient to fully understand the comprehensive role of these professionals and to recognize their importance.

Concerning the willingness to use medical interpreter services, both a survey by the Ministry of Health, Labour and Welfare [4] and research by Hamai et al. [6] showed that most hospitals with experience using medical interpreters responded that the experience was generally positive and that they would like to use specialized interpreters if the service were reimbursable under the national medical fee schedule. Despite medical interpreters not being easily accessible within the current Japanese system, it is expected that increased usage among health care professionals will boost providers’ willingness to use medical interpreter services and lead to the wider dissemination of these services. Therefore, we consider that promoting the use of medical interpreters among health care professionals is crucial.

Limitations and importance

The present study has several limitations. First, the number of respondents was strictly limited to 100, and the response rate was extremely low at 0.60%. Consequently, the findings cannot be generalized to the broader population of health care professionals. Second, the question regarding the perceived need for medical interpreting was framed simply as whether the respondent felt that a medical interpreter was necessary when treating foreign patients. Therefore, it cannot be conclusively stated whether health care professionals fully understood the interpreter's original role and appropriate usage when responding.

Furthermore, the analysis focused on 88 respondents who had experience treating foreign patients out of the total 100 participants. It is possible that the experience of treating foreign patients itself influenced respondents' views on the perceived need for medical interpreting. Future research should therefore expand the sample size and examine whether differences exist based on the presence or absence of foreign patient care experience.

Notwithstanding these limitations, the current study is considered important because its findings help clarify the factors associated with the perceived need for medical interpreting among health care professionals who have experience providing care for foreign patients.

## Conclusions

The results of this study revealed that age and willingness to use medical interpreter services were associated with the perceived need for medical interpreting among health care professionals with experience treating foreign patients. Furthermore, age, length of work experience, profession, and prior experience using medical interpreters were found to influence the willingness to use medical interpreter services.

It is anticipated that an increase in the number of health care professionals with prior experience using medical interpreters will lead to a greater willingness to use medical interpretation services among these professionals, thereby contributing to the broader dissemination of medical interpreting. Therefore, we believe it is crucial to promote the use of medical interpreters among health care providers.
